# Could lockdown increase the incidence of eating disorders?

**DOI:** 10.1192/j.eurpsy.2021.595

**Published:** 2021-08-13

**Authors:** M. Jiménez Cabañas, A. García Carpintero, V. Pérez Navarro, M.R. Pérez Moreno

**Affiliations:** Psychiatry And Mental Health, Hospital Clínico San Carlos, Madrid, Spain

**Keywords:** eating disorder, adolescent, lockdown

## Abstract

**Introduction:**

Spanish Governmen declared state of emergency in March 2020 to prevent coronavirus COVID-19 from spreading. During September and October 2020, at Child and Adolescent Psychiatry Unit we have attended patients who presented altered eating behaviors whose onset was during lockdown. We report a series of seven cases of adolescent girls between the ages of 11 and 16, who had no previous history of mental illness. During lockdown, they have presented restriction of food and increased physical exercise, with weight loss. Some of these patients have also presented food binges and purging behaviors.

**Objectives:**

Review the impact of lockdown on eating behavior, specially on weight loss.

**Methods:**

Literature review of scientific papers searching in Pubmed.

**Results:**

There are articles that study the variations in eating habits and exercise ocurred during confinement. Most focus on two trends: on the one hand, increased intake and the tendency to a more sedentary life; on the other hand, the worsening of people with a previous diagnosis of eating disorder. However, there is a third trend for which there are few studies: the new appearance of restrictive eating behaviors, together with increased physical exercise, bingeing and purging. This is the case of the patients we present. These studies describe as a possible cause of these alterations that confinement is a novel situation, which generates stress, social isolation, boredom, anxiety and a feeling of loneliness, which can influence self-concept and eating behaviors.

**Conclusions:**

Lockdown has favored a change in eating habits and exercise. More studies are needed on new-onset eating disorders.

